# Methodological comparison of cancellation versus two-way choice spatial attention tests in humans and dogs

**DOI:** 10.3389/fvets.2023.1264151

**Published:** 2023-10-13

**Authors:** Anna Kis, Eszter Radics, Henrietta Bolló, József Topál

**Affiliations:** ^1^Institute of Cognitive Neuroscience and Psychology, Research Centre for Natural Sciences, Budapest, Hungary; ^2^ELTE-HUNREN NAP Comparative Ethology Research Group, Budapest, Hungary

**Keywords:** canine model, visual neglect, spatial cognition, dog, side bias

## Abstract

**Introduction:**

Behavioural problems in family dogs are amongst the leading reasons for relinquishment to shelters which adversely affects animal welfare. Recent research suggests that certain problematic behavioural patterns might be analogous to human psychiatric disorders. Veterinary diagnosis of such conditions, however, is scarce, probably due to the lack of appropriate measurement tools. The current study focuses on dog behaviour resembling the human hemispatial neglect condition, which manifests itself as a deficit in attention to and awareness of one side of the space.

**Methods:**

Healthy human subjects (N = 21) and adult family dogs (N = 23) were tested with tools aimed to measure spatial attention. Tests administered to humans included validated paper and pencil neuropsychological tools to assess hemispatial neglect (cancellation tasks), as well as the canine version of that task (visuo-spatial search task). Dogs were tested with the same visuo-spatial search task as well as a two-way choice task.

**Results:**

Results show that both in case of dogs and humans the visuo-spatial search task detects individual variation in subjects’ side preferences. However, subjects’ performance in the different tasks were not related.

## Introduction

Family dogs are amongst the most popular pets worldwide (1, 2) with a considerable economical and personal impact on human societies. Whilst there is a growing body of knowledge about dog behaviour and cognition (3), the veterinary conditions paralleling human psychiatric disorders are vastly understudied, despite the fact that the dog has been suggested to suffer from many human-like diseases (4, 5). The few studies focusing on this research line include evidence on the presence of symptoms resembling Obsessive Compulsive Disorder (6, 7), Attention Deficit Hyperactivity Disorder (N (8)) and autism (9, 10). These results, however, are of studies from recent years only and do not go beyond a couple of papers. This is especially surprising, since behavioural problems are often reported by owners as those most devastating for every-day life and well-being (11), and unwanted behaviours are amongst the most common reasons for relinquishing dogs to animals shelters (12). Perhaps not surprisingly, in addition to aggressive behaviours (which are the leading reason for animal relinquishment to shelters) other – not directly harmful – behaviours such as being afraid of various things, demanding too much attention and being disobedient are commonly mentioned by owners as a reason to get rid of their dogs (12). Cognitive / psychiatric-like symptoms, amongst which the most commonly studied one is canine cognitive dysfunction, are also perceived as an increased burden by dog owners (13). This in addition to the fact that such conditions prevent the caregivers from living a full life, constitutes a serious animal welfare issue as long as they cannot be properly treated and / or prevented. The present study aims to give empirical data on the currently available behavioural tools to measure one such human-like condition, namely the behavioural pattern resembling hemispatial neglect.

Recently it has been suggested (14) that dogs’ side bias behaviour, which is often reported in cognitive experiments, shows parallels to human hemispatial neglect. The psychiatric condition of hemispatial neglect is characterised by a deficit in attention to and awareness of the contralesional hemispace following a brain injury (15). The behavioural manifestation of unilateral spatial neglect in everyday life includes the patient bumping into objects on the neglected side, ignoring the food on one side of the plate, difficulties in dialling numbers, watching TV, playing social games and not responding to people situated on their neglected side. There are also a couple of documented cases of dogs showing hemispatial neglect including a citizen science example published on a personal blog^1^ where the dog ignored food on half of the plate, as well as a veterinary case study (16) where the dog only responded to stimulation coming from one side. The study of Bolló et al. (14) applied a spontaneous two-way-choice task, and tested a group of dogs with previous side bias history (they had previously shown 100% bias to left or right in other two-way choice tasks). It was found that the majority of the subjects retained their side bias suggesting that the phenomena is stable across time and situations. Furthermore, dogs relied on an egocentric reference frame in their choices, similarly to human patients.

Whilst these indirect parallels are an important first step, there are still methodological problems that prevent the application of such behavioural tests in veterinary practice. First and foremost, to date there is no direct comparison between dogs and humans’ neglect-like behaviour. In human patients, hemispatial neglect is typically assessed *via* paper and pencil methods, such as the clock-drawing task (17), line bisection or the cancellation test (18). In these cases, human subjects have to make a drawing, or cross out certain objects in an array of distractors. These tests are obviously not compatible with dogs’ motor skills. There has been an effort to develop a visuo-spatial search task matching the cancellation test that can be performed with dogs (19); this is essentially a food detection task with a grid of possible hiding places and was successfully implemented by the authors.

A further problem with the veterinary applicability of testing human-like psychiatric conditions in dogs, is that standardised information regarding the normal range versus the pathological dog behaviour is mostly lacking see, e.g., Csibra et al. (20) for a similar argument. Thus in addition to the approach used in Bolló et al. (14) tests need to be performed on healthy subjects as well.

It is important to note that hemispatial neglect is a debilitating condition making human patients’ everyday life more challenging due to patients failing to notice objects or people on one side (15) and thus having difficulties, e.g., crossing the road or navigating in the environment. An important criteria for side bias in dogs is that it leads to disadvantageous choices: subjects with a side bias receive less food reward in cognitive tests. For example in a pointing-following test (21) where dogs generally perform above chance (receiving a food reward for “good” choices, and receiving no reward for “bad” choices), subjects with a side bias will perform merely at chance. This differentiation needs to be made from behavioural and brain lateralisation – a natural phenomenon wide-spread throughout the vertebrate taxa (22) –, which on the contrary is argued to have adaptive functions. There is also ample literature on dogs’ lateralised response to different (e.g., emotional) stimuli (23), which again is a research line different from disadvantageous attentional biases.

In the current paper we first aim to test whether humans’ performance in the validated pen and paper cancellation tests matches the visuo-spatial search task claimed to be the canine cancellation test. Next we will test dogs in both the canine cancellation (visuo-spatial search) task as well as in two-way choice tests where parallels between dog side bias and human hemispatial neglect had been shown.

## Method

### Ethics statement

Research on dogs were approved by the National Animal Experimentation Ethics Committee (Ref No. XIV-I-001/531–4-2012). The research on dogs was done in accordance with the Hungarian regulations on animal experimentation and the guidelines for the use of animals in research described by the Association for the Study Animal Behaviour (ASAB). All owners volunteered their dog to participate in the study and they gave written informed consent.

Research on human subjects was approved by the National Research Ethics Committee (PE/EA/55–4/2019), by the Research Ethics Committee of Eötvös Loránd University and was carried out in accordance with the Declaration of Helsinki. All participants volunteered to take part in the study and were asked to fill out an informed consent form in accordance with an Institutional Review Board-approved protocol. They were aware of their right to withdraw from participation at any time of the procedure.

### Subjects

Dog participants were N = 23 healthy subjects aged between 1–12 year-old (mean ± SE = 5.4 ± 3.1 years), including 11 females (9 neutered) and 12 males (4 neutered), of 11 different breeds and 12 mongrels.

Human participants included N = 21 healthy young adults aged 22–36 year-old (mean ± SE = 26.8 ± 3.03 years), including 11 females and 10 males, all right-handed.

### Procedure – dogs

Dogs completed a visuo-spatial task, which is considered the canine version of the cancellation test (19), as well as a two-way choice task, which has been used to show parallels between canine side bias and human hemispatial neglect (14).

**Figure 1:**
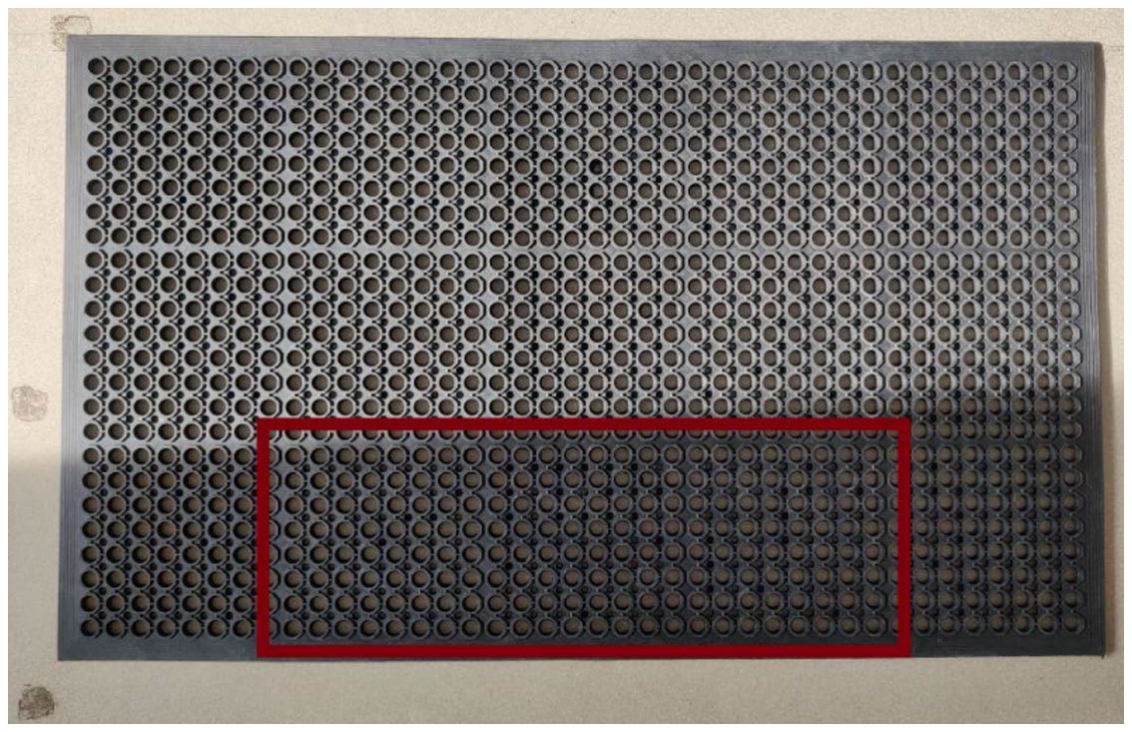
Rubber matt apparatus used for the visuo-spatial test. The red rectangle highlights the area baited in the test.

The visuo-spatial task used a rubber matt apparatus with an array of circular holes in it (2.5 cm diameter; Figure 1). The target area consisted of 8 × 8 holes for the “left,” 8 × 8 holes for the “middle” and 8 × 8 holes for the “right” (total of 86 cm × 28 cm for the three target areas). The owner sat on a chair facing the middle of the matt with the dog in between his/her legs (positioned exactly in the middle). The experimenter occluded the apparatus with a paravan, then hid 4–4-4 targets pseudo-randomly within the left-middle-right areas. The targets were dry dog food pallets, which the subjects could eat. After the experimenter removed the paravan, dogs were allowed to search for the food without giving any instruction or orienting them left / right. The pre-set time limit for the test was 5 min, but all subjects were quicker to complete the trials. The test was repeated for a total of six times.

For the two-way choice task two flower pots were baited with a dry dog food pellet each. The owner was asked to sit on a chair placed equidistant (1.5 m) from the two prospective hiding locations and made the dog sit in front of him/her Whilst holding the dog’s collar. The experimenter asked the owner not to let the dog free until she signs with a nod. After this, 6 trials were carried out. The experimenter crouched down, and placed the two baited containers simultaneously on the floor at an arm-length distance from herself. Then the experimenter stood up and stepped back looking straight ahead above the head of the owner. Finally she nodded to the owner and the dog was allowed to make a choice.

Both tests were carried out in a 5.27 m × 6.23 m behavioural laboratory equipped with a four-camera system (Logitech HD Pro Webcam C920). The order of the two tests was counterbalanced across subjects and were carried out consecutively with a short in-between break as needed (e.g., for the subject to drink some water).

### Procedure – humans

Human subjects completed two previously validated paper & pencil cancellation tests (see below) as well as the above described visuo-spatial task, which is considered the canine version of the cancellation test. This allowed us to compare human subjects’ performance to dogs’ in the visuo-spatial task as well as a comparison between the visuo-spatial task and previously validated cancellation tests.

**Figure 2:**
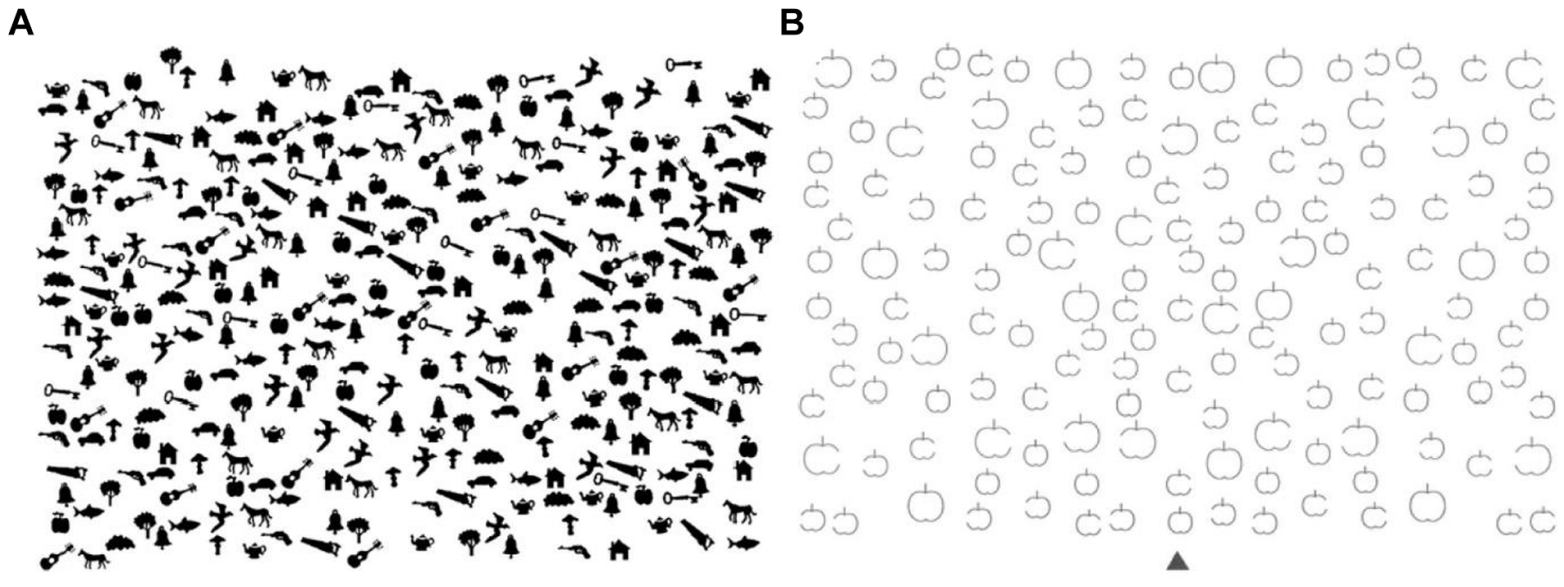
Paper & pencil cancellation tests used in the current study. On the left **(A)** is the Bells test, where subjects need to find the total of 35 bells. On the right **(B)** is the Apples test where subjects need to find the total of 50 intact apples.

The cancellation tests were the Bells test and the Apples test, both of which have been widely used in clinical settings to diagnose neglect syndrome. The Bells test (24) contains 315 stimuli, including 35 targets and 280 distractors (Figure 2A). Targets are pseudo-randomly positioned in 7 columns, with 5 targets in each. For the purpose of the analysis three columns are considered “left,” one column is “middle” and three columns are “right.” This test is used to diagnose egocentric neglect *via* measuring the number and position of omitted targets. The Apples test (25) presents 150 stimuli, including 50 targets and 100 distractors (Figure 2B). The targets are pseudo-randomly positioned in a 2 rows by 5 columns with an equal number of targets in each cell. For the purpose of the analysis 2 columns are considered “left,” one column is “middle” and two columns are “right”; the up and down rows were not separated for the present experiment. This test is used to diagnose allocentric neglect, as some of the apples miss a bite on the left side, Whilst others on the right side.

The visuo-spatial task (which is considered the canine version of the cancellation test) used a rubber matt apparatus (same as above described for dogs) with an array of circular holes in it (2.5 cm diameter). The target area consisted of 8 × 8 holes for the “left,” 8 × 8 holes for the “middle” and 8 × 8 holes for the “right,” and 4–4-4 targets were pseudo-randomly hidden in each. The targets were the same dry dog food pallets as above, but human subjects had to collect them by hand, and of course they did not eat them. Subjects were asked to turn their back Whilst the experimenter hid the targets. Then they turned back, crouching on the floor and positioned exactly to the middle line of the apparatus, and were instructed to collect the targets. Subjects were not restricted in whether they used the left / right or both hands to reach for the targets. The test was repeated for a total of six times.

The visuo-spatial test was carried out in the same 5.27 m × 6.23 m behavioural laboratory as in case of dogs. For the paper & pencil tests subjects were seated to a table in an adjacent office. The order of the two tests as well as the order of the two cancellation tasks was counterbalanced across subjects and were carried out consecutively with a short in-between break as needed (e.g., for the subject to drink some water).

### Data analysis

In all tests (both for dogs and humans) the analysis focused on left versus right targets. For the statistical analysis the number of “left” choices were used, due to human subjects group-level bias to solve paper & pencil tasks from left to right (in reading direction, see Results). For all tests we analysed the first N number of choices, N being the maximum possible number of left choices that can be made. Thus for dogs: the number of “left” choices in the two-way choice task (6 trials) and the first 24 choices (first 4 choices × 6 trials) in the visuo-spatial test. For humans: first 20 choices (Apples test), first 15 choices (Bells test) and first 24 choices (first 4 choices × 6 trials; visuo-spatial test).

Spearman correlations were carried out within both dog and human data to find any association in left bias between tests. Then we compared male and female as well as neutered and non-neutered dogs regarding left bias (Wilcoxon test) and tested the relationship between dogs’ age and left bias (Spearman correlation). Male and female human subjects were also compared regarding left bias (Wilcoxon test); age effects were not analysed in humans due to the narrow age-range of the subjects (all young adults).

Additionally, in each test the above described outcome measure (number of “left” choices) was compared to the subjects’ first choice. For dogs a Mann–Whitney test was used to compare the number of “left” choices in the two-way choice task between subjects who first chose left versus right; and a Kruskal Wallis test was used to compare the number of “left” choices in the visuo-spatial test between subjects who first chose left versus middle versus right. For humans, data from the Apples and the Bells tests could not be analysed using first choice as a grouping variable (as almost all subject had a “left” first choice; see result). A Kruskal Wallis test was used to compare in human subjects the number of “left” choices in the visuo-spatial test between subjects who first chose left versus middle versus right.

## Results

### Dog subjects – descriptive data

Dogs performed at ceiling in the visuo-spatial search task: out of the N = 23 subjects N = 21 (91%) correctly found all 24 food pellets on the left side, N = 21 (91%) correctly found all 24 food pellets in the middle, and N = 20 (87%) correctly found all 24 food pellets on the right. One dog made 5 errors (1 omission in the middle and 3 omissions on the right), one dog made 6 errors (1 omission on the left and 5 omissions on the right), and one dog made 7 errors (3 omissions on the left, 3 omissions in the middle and 1 omission on the right). Dogs first choices were mostly made in the middle region (N = 13; 57%), Whilst some started on the left (N = 4; 17%) and on the right (N = 6; 26%) regions as well.

In the two-way choice task, N = 10 dogs (43%) made their first choice to the left, and N = 13 (57%) dogs made their first choice to the right. There were N = 4 dogs that made 6/6 left choices, N = 5 that made 6/6 right choices, Whilst the remaining N = 14 made both left and right choices.

### Dog subjects – relationship between the different tests

For comparability with human data (see below) side-bias in the subjects’ search pattern was analysed by looking at the number of “left” choices in the two-way choice task (6 trials) and the first 24 choices (first 4 choices × 6 trials) in the visuo-spatial test, respectively. There was no correlation between left bias in the two tests (r = 0.144, *p* = 0.513).

**Figure 3:**
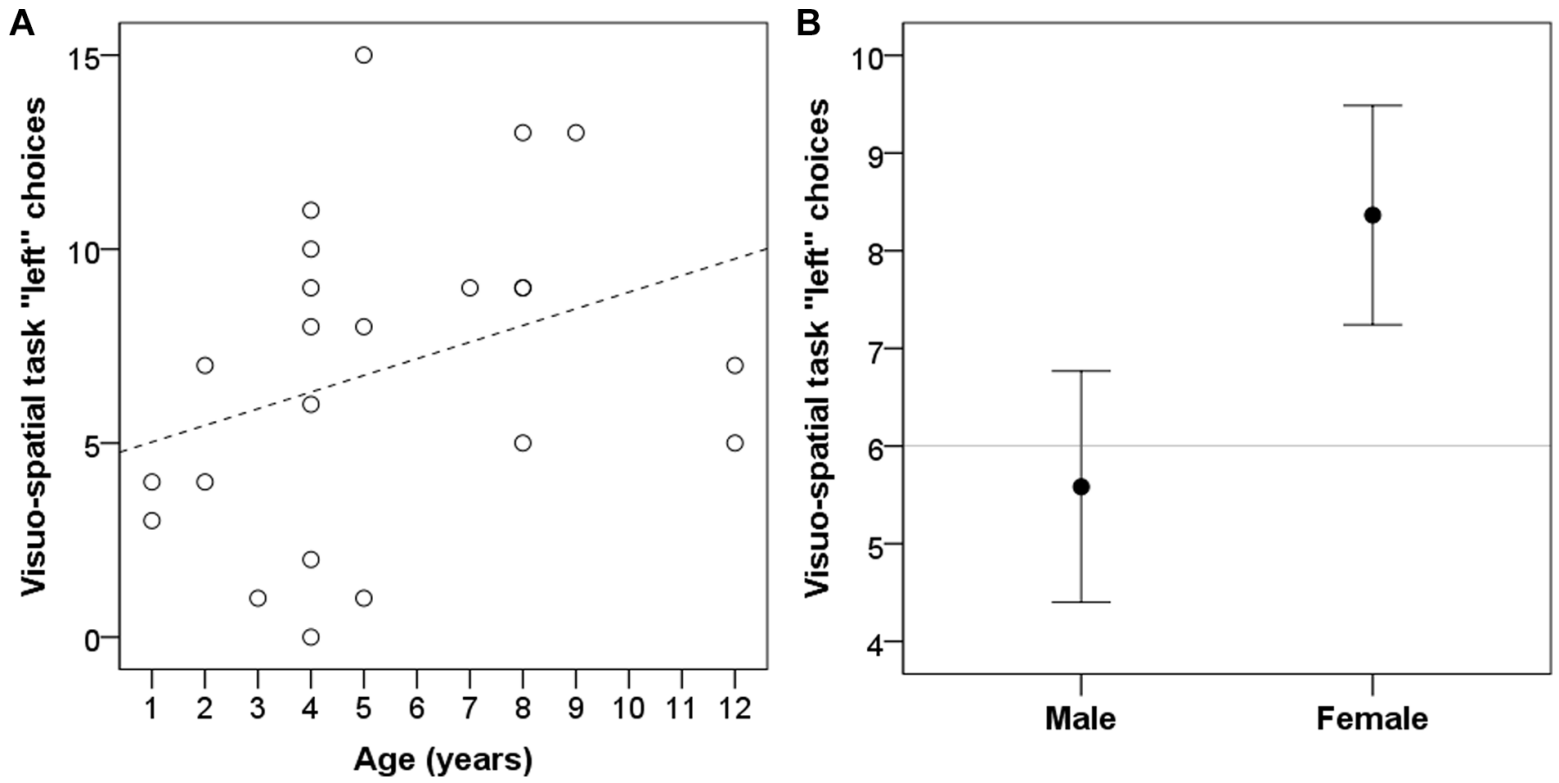
Dogs’ left bias (number of left choices) in the visuo-spatial task was significantly influenced by subjects’ age **(A)** and was different between males and females **(B)**. Grey line represent chance level.

**Figure 4:**
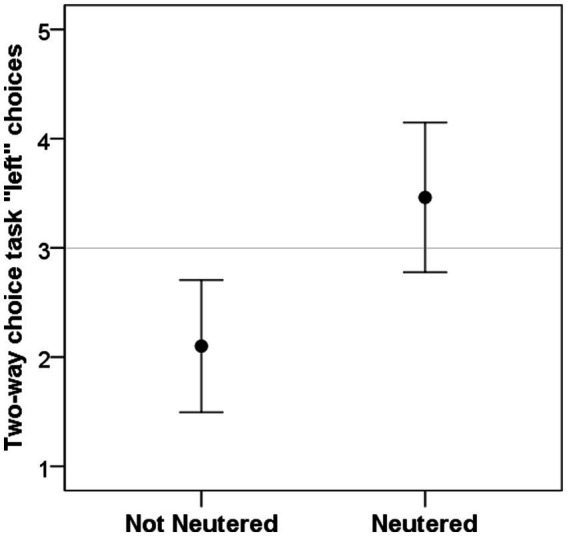
Dogs’ left bias (number of left choices) in the two-way choice task was significantly different in neutered individuals. Grey line represent chance level.

Dogs’ age was significantly correlated with left bias in the visuo-spatial search task (r = 0.418, *p* = 0.047; Figure 3A), but not in the two-way choice task (r = 0.139, *p* = 0.526). Dogs’ sex had a significant influence on left bias in the visuo-spatial search task (W = 102, *p* = 0.009; Figure 3B) with females being making more left choices; but had no effect on the two-way choice task (W = 118.5, *p* = 0.115). Neutering status of the dog, on the other hand, significantly influenced left bias in the two-way choice task (W = 84.5, *p* = 0.027; Figure 4) with neutered dogs making more left choices; but had no influence on the visuo-spatial search task (W = 100, *p* = 0.209).

### Dog subjects – first choice

In the two-way choice task, dogs (N = 10) who chose left in the first trial had a significantly higher total number of “left” choices compared to those (N = 13) who first chose right (U = 15; *p* = 0.002). However, in the visuo-spatial task no such difference could been found (Chi^2^_(2)_ = 3.001, *p* = 0.223) between dogs that first chose left (N = 4) / middle (N = 13) / right (N = 6), nor between those dogs alone that first chose left versus right (U = 4.5, *p* = 0.107).

### Human subjects – descriptive data

Human subjects performed at / near ceiling both in the paper & pencil tests and the visuo-spatial search test. In the Apples test, out of the N = 21 participants N = 17 (81%) correctly found all 20 targets on the left side, N = 20 (95%) correctly found all 10 targets in the middle, and N = 14 (67%) correctly found all 20 targets on the right side; the maximum number of errors was 4 / subject. In the Bells test, out of the N = 21 participants N = 16 (76%) correctly found all 15 targets on the left side, N = 20 (95%) correctly found all 5 targets in the middle, and N = 19 (90%) correctly found all targets on the right side; the maximum number of errors was 3 / subject. In the visuo-spatial search test out of the N = 21 participants N = 20 (95%) correctly found all 24 targets on the left, N = 21 (100%) correctly found all 24 targets in the middle, and N = 19 (90%) correctly found all 24 targets on the right; the maximum number of errors was 1 / subject.

In both paper & pencil tests human subjects predominantly searched from left to right, following the same direction as we read. Out of the N = 21 subjects only N = 3 (14%) made a non-left first choice in the Apples test, and N = 2 (9.5%) in the Bells test. In the visuo-spatial search task, however, left – middle – right first choices were evenly distributed: N = 6 (29%) left first, N = 8 (38%) middle first, N = 7 (33%) right first.

### Human subjects – relationship between the different tests

**Figure 5:**
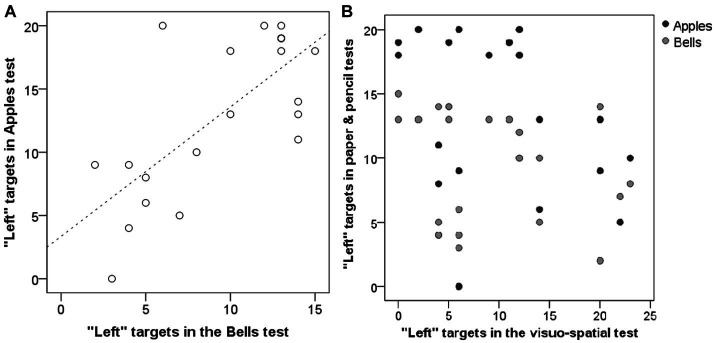
Relationship between human subjects’ left bias in the Bells and Apples test **(A)** as well as between the visuo-spatioal test and the two paper & pencil tests **(B)**.

Side-bias in the subjects’ search pattern was analysed by looking at the number of “left” answers in their first 20 choices (Apples test), first 15 choices (Bells test) and first 24 choices (first 4 choices × 6 trials; visuo-spatial test) respectively. Left bias in the two paper & pencil tests (Apples, Bells) was significantly correlated (r = 0.589, *p* = 0.005; Figure 5A). However, left bias in the visuo-spatial search task was not related to any of the paper & pencil tests (Apples: r = −0.260, *p* = 0.256; Bells: r = −0.313, *p* = 0.167; Figure 5B).

No difference was found between males and females in their left bias in any of the tasks (Apples: W = 114.5, *p* = 0.646; Bells: W = 104, *p* = 0.227; Visuo-spatial: W = 97, *p* = 0.090).

### Human subjects – first choice

Human subjects in the paper & pencil tests predominantly made left first choices (N = 18 / 21 in the Apples test; N = 19 / 21 in the Bells test); thus first choices could not be compared statistically to the total number of “left” choices. In the visuo-spatial test, however, there was a significant difference in the total number of left choices between those subjects who first chose left / middle / right (Chi^2^_(2)_ = 9.162, *p* = 0.010): those who made a “left” first choice made significantly more total left choices, than those who first chose middle (U = 6.0, p = 0.010) or right (U = 4.5, *p* = 0.011), Whilst there was no difference between the latter two (U = 19.0, *p* = 0.774).

## Discussion

The current study tested different visuo-spatial tasks in both dogs and humans with the aim of establishing a comparative framework to study hemispatial neglect-like side biases. Our findings are mixed regarding the reliability and potential veterinary application of the tasks tested. We have found that both canine tasks are easy to administer – and are thus suitable for the use in veterinary settings. However, a study involving more subjects (both healthy as well as post-stroke dogs) would be needed to establish standard thresholds for veterinary diagnosis. An additional important finding is that dogs’ first choice in the two-way choice task and human’s first choice in the visuo-spatial task is a significant predictor of total “left” choices. This means that the tests could potentially be shortened to a single trial each, which would further ease their administration in veterinary settings. This is in line with findings on other species (e.g., cats: (26)) as well. We should note, however, that for the visuo-spatial tasks no significant association was found between first choice and total score in case of dogs. Whilst this is likely due to the predominant “middle” first choices (and thus the low subject numbers for left versus right choice), the test needs to be carried out on a larger sample (with more left- and right first-choosers) before the validity of the first choice can be confirmed.

In humans we have shown, as expected, that healthy young subjects perform at ceiling in paper & pencil cancellation tests used with clinical patients. The tests (‘Apples’ test and ‘Bells’ test) still yielded meaningful data with between-individual variability when focusing on the side-bias aspect of the responses. The ceiling performance of healthy subjects in a test designed for neglect patients is not surprising as they were given the same time to complete the search. For the present analysis we only used subjects’ first few answers, which is essentially equal to a stricter time limit if we consider the remaining targets as omissions. Using this method, subjects’ left bias in the two paper & pencil tasks were significantly related confirming that these are reliable measures. The left bias found in the present study confirms previous reports of human cancellations tests (27, 28), meaning that in paper and pencil tests subjects find stimuli on the left first. This pattern presumably aligns with the left-to-right reading direction, although findings on, e.g., Arabic populations are mixed: some did find a right bias in visual scanning, as would be expected based on the reading direction (29), but others studying Arabic subjects (30) found a left bias similar to our finding, although only in the 63% of the subjects, which is lower compared to the present results (‘Apples’ 84%, ‘Bells’ 91%). Yet others (31) have found bidirectional search patterns in bilingual subjects with one language reading from left to right, whereas the other language reading from right to left. Illiterate subjects have been shown to manifest a disorganised visual search strategy (32). So taken together it does seem that culturally learned reading direction influences search pattern in paper and pencil cancellation tests. On the other hand, performance in the visuo-spatial task (which was designed to mimic the dog version of the cancellation test) was not related to performance in either of the paper & pencil cancellation tests. However, considering subjects’ left biases in the ‘Apples’ and ‘Bells’ tests this finding does not invalidate the visuo-spatial search task, which was free from such a strong left bias and showed a more even distribution between left and right searches as could be expected from healthy subjects.

For the dog subjects we also found a near-ceiling search performance with meaningful variance in their left / right biases. Furthermore, no relationship was found between left bias in the two-way choice task versus the visuo-spatial search task. This suggests that the two tests measure different aspects of side bias and spatial cognition, despite the fact that both had been validated to some extent. Spontaneous two-way choice tasks have been shown to be a reliable measure of side bias (14), and they have also been used to confirm magnetic alignment in dogs (33). The visuo-spatial search task was shown to be related to left / right paw use in a Kong test (19). Dogs’ paw preference was not assessed in the present paper – mainly because dogs used their muzzles / full body to complete their task, so paw preference was not a direct confounding factor for the outcome measure. Thus based on the current dataset we cannot provide results for or against the relationship between visuospatial attention and paw preference. It is important to note, however, that the wide variety of available tests measuring such motor laterality in dogs (e.g., paw that the dogs give on command, paw used to hold a chew or a Kong, paw preference during locomotion or when stepping on the stairs, etc) have been shown to yield inconsistent results across tasks (34). Thus these test likely measure slightly different forms of motor laterality, and at least some of these can potentially be related to the here used side bias tests – e.g. head turning responses (35) or paw used to remove an adhesive tape from the nose (36) might relate to variability in muzzle use which future research applying the current tests needs to take into account. The results of the present study align nicely with the inconsistencies reported on other species and across tasks (37–39), as it was similarly found that the two side bias measures currently investigated in dogs were not related. It is further argued (40) that task complexity and other factors related to the specific testing situation (e.g., tastiness of the item to be retrieved) also affect lateralized behaviour.

Here we found that dogs’ side bias in the visuo-spatial task is influenced by their age and sex. This further argues for the validity of the test, since old dogs have been previously shown to be more prone to develop side bias (41). The effect of sex was in the opposite direction compared to previous reports on paw preference (36, 42), as we found that females were more left-biased, as opposed to the right paw-preference reported. Others (e.g., (43–45)), however, have not found any sex-differences in laterality.

Despite behavioural parallels between human hemispatial neglect and dog side bias (e.g., (14)) the underlying brain mechanisms are likely to differ in the two species. It has been shown for example in an fMRI based study that lexical processing in dogs is localised in the right hemisphere, contrary to humans (46). Such brain level differences, however, do not invalidate the use of animal models in psychiatric research, and dogs are continuously considered as a valid parallel to human neuro-cognition (47).

A further point to be considered for the here presented results is that due to the method validation aspect of the current study, it involved healthy subjects (both dogs and humans). For humans it has been reported, that – as in case of most psychiatric disorders – there is a continuum between unilateral spatial neglect symptoms and normal behaviour (48). The so called pseudoneglect has been described in clinically healthy patients, being a mild form of sidedness, manifesting, e.g., in bumping into objects on the left side with a greater probability than on the right side (49). This sub-clinical attentional bias has also been connected to, e.g., eye-blink rate in humans (50). Such connections are yet to be established between neurologically healthy dogs with side bias and post-stroke dogs showing hemispatial neglect symptoms.

In conclusion, we have shown here, that an easy to carry out behavioural test, such as the visuo-spatial search task used here, can be used to detect individual differences in dogs’ (and humans’) side-bias. Since the currently studied subjects were all neurologically healthy, further research needs to determine if such individual differences underlie, e.g., predisposition for hemispatial neglect-like symptoms later on. Additionally the treatment and prognosis – known to vary considerably in case of human neglect patients (51) – needs to be studied in dogs as well.

## Data availability statement

The raw data supporting the conclusions of this article will be made available by the authors, without undue reservation.

## Ethics statement

The studies involving humans were approved by National Research Ethics Committee, Research Ethics Committee of Eötvös Loránd University. The studies were conducted in accordance with the local legislation and institutional requirements. The participants provided their written informed consent to participate in this study. The animal studies were approved by National Animal Experimentation Ethics Committee. The studies were conducted in accordance with the local legislation and institutional requirements. Written informed consent was obtained from the owners for the participation of their animals in this study.

## Author contributions

AK: Conceptualization, Data curation, Formal analysis, Funding acquisition, Methodology, Supervision, Visualization, Writing – original draft, Writing – review & editing. ER: Formal analysis, Investigation, Writing – review & editing. HB: Conceptualization, Methodology, Supervision, Writing – review & editing. JT: Conceptualization, Funding acquisition, Methodology, Supervision, Writing – review & editing.

## Footnotes

^1^See the related youtube video: https://youtu.be/hHo-y7WJIlU
